# Crystal structure of (*E*)-2-cyano-3-(12-methyl-12*H*-benzo[*b*]pheno­thia­zin-11-yl)acrylic acid

**DOI:** 10.1107/S1600536814018388

**Published:** 2014-08-20

**Authors:** Motonori Watanabe, Tatsumi Ishihara

**Affiliations:** a International Institute for Carbon-Neutral Energy Research (WPI-I2CNER), Kyushu University. 744 Motooka, Nishi-ku, Fukuoka 819-0395, Japan; b Department of Applied Chemistry, Kyushu University, 744 Motooka, Nishi-ku, Fukuoka 819-0395, Japan

**Keywords:** crystal structure, benzo[*b*]pheno­thia­zine derivative, inversion dimers, hydrogen bonding, π–π stacking

## Abstract

In the title compound, C_21_H_14_N_2_O_2_S, a donor–acceptor type of benzo[*b*]pheno­thia­zine (bpz) derivative, the thia­zine ring adopts a boat conformation and the bond-angle sum at the N atom is 360.0°. The dihedral angle between the benzene ring and the naphthelene ring system fused to the thia­zine ring is 32.76 (5)°. In the crystal, carb­oxy­lic-acid inversion dimers linked by pairs of O—H⋯O hydrogen bonds generate *R*_2_^2^(8) loops. Aromatic π–π stacking [shortest centroid–centroid separaton = 3.5242 (13)Å] consolidates the structure and very weak C—H⋯O and C—H⋯N inter­actions also occur.

## Related literature   

For related structures, see: Bell *et al.* (1968 ▶), van de Waal & Feil (1977 ▶), Sun *et al.* (2004 ▶); Harrison *et al.* (2007 ▶). For applications of the title compound in dye-sensitized solar cells, see: Watanabe *et al.* (2014 ▶).
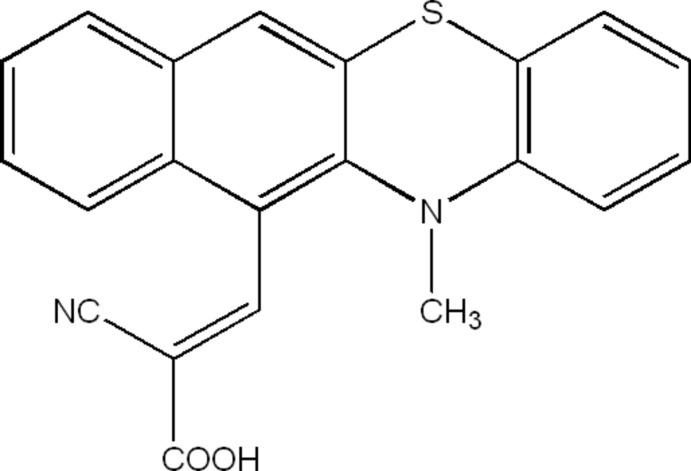


## Experimental   

### Crystal data  

C_21_H_14_N_2_O_2_S
*M* *_r_* = 358.40
Triclinic, 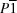

*a* = 6.7915 (17) Å
*b* = 9.196 (3) Å
*c* = 13.941 (3) Å
α = 95.831 (13)°
β = 101.214 (10)°
γ = 90.850 (15)°
*V* = 849.1 (4) Å^3^
*Z* = 2
Mo *K*α radiation
μ = 0.21 mm^−1^
*T* = 123 K
0.40 × 0.40 × 0.15 mm

### Data collection  

Rigaku R-AXIS RAPID CCD diffractometer
14164 measured reflections
3877 independent reflections
3663 reflections with *I* > 2σ(*I*)
*R*_int_ = 0.020

### Refinement  

*R*[*F* ^2^ > 2σ(*F* ^2^)] = 0.036
*wR*(*F* ^2^) = 0.100
*S* = 1.06
3877 reflections
290 parameters
H-atom parameters constrained
Δρ_max_ = 0.29 e Å^−3^
Δρ_min_ = −0.40 e Å^−3^

### 

Data collection: *RAPID-AUTO* (Rigaku, 1998 ▶); cell refinement: *RAPID-AUTO*; data reduction: *RAPID-AUTO*; program(s) used to solve structure: *SIR97* (Altomare *et al.*, 1999 ▶); program(s) used to refine structure: *SHELXL2014* (Sheldrick, 2008 ▶); molecular graphics: *ORTEP-3 for Windows* (Farrugia, 2012 ▶); software used to prepare material for publication: *Yadokari-XG 2009* (Wakita, 2001 ▶; Kabuto *et al.*, 2009 ▶) and *POV-RAY* (Persistence of Vision Team, 2004 ▶).

## Supplementary Material

Crystal structure: contains datablock(s) General, I. DOI: 10.1107/S1600536814018388/hb7264sup1.cif

Structure factors: contains datablock(s) I. DOI: 10.1107/S1600536814018388/hb7264Isup2.hkl

Supporting information file. DOI: 10.1107/S1600536814018388/hb7264Isup3.cml

**Fig. 1.**
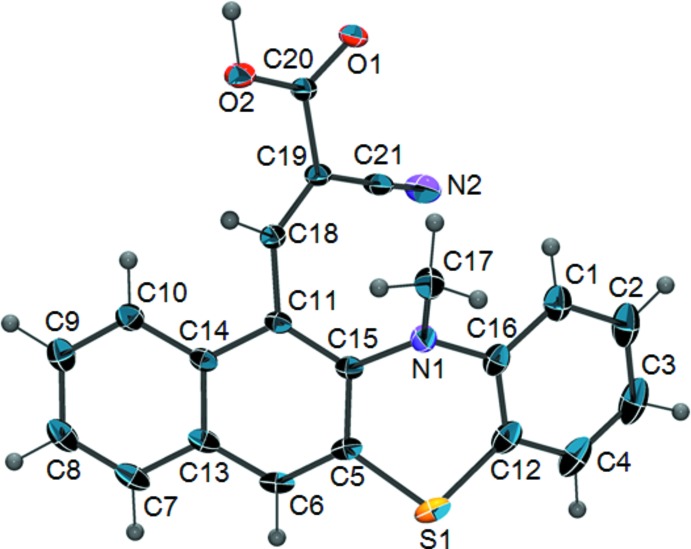
The molecular structure of C21H14N2O2S with displacement ellipsoids drawn at the 50% probability level.

21 14 2 2 . DOI: 10.1107/S1600536814018388/hb7264fig1.tif
The mol­ecular structure of C_21_H_14_N_2_O_2_S with displacement ellipsoids drawn at the 50% probability level.

**Fig. 2.**
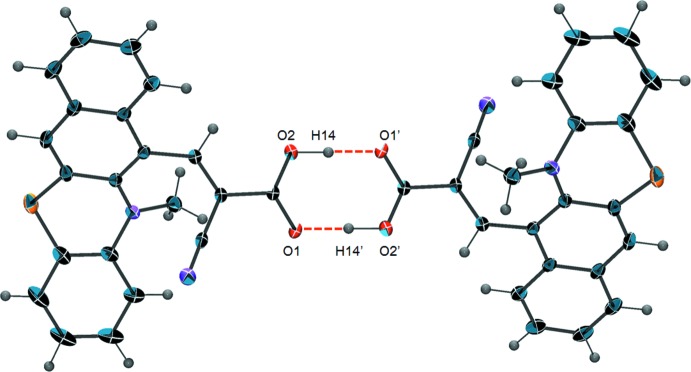
Intermolecular hydrogen bonding O1—H14—O2' (1.520 Å) in the two molecule structure of carboxylic acid moiety.

. DOI: 10.1107/S1600536814018388/hb7264fig2.tif
Inter­molecular hydrogen bonding O1—H14—O2′ (1.520 Å) in the two mol­ecule structure of carb­oxy­lic acid moiety.

**Fig. 3.**
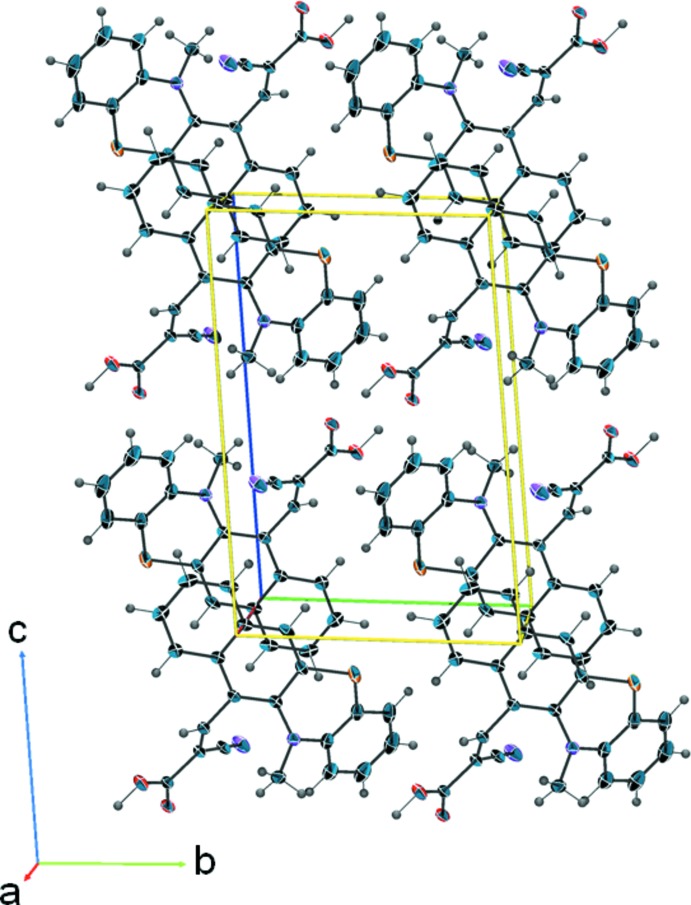
Packing diagram of C21H14N2O2S.

21 14 2 2 . DOI: 10.1107/S1600536814018388/hb7264fig3.tif
Packing diagram of C_21_H_14_N_2_O_2_S.

CCDC reference: 1018980

Additional supporting information:  crystallographic information; 3D view; checkCIF report

## Figures and Tables

**Table 1:** Hydrogen-bond geometry (Å, °)

*D*—H⋯*A*	*D*—H	H⋯*A*	*D*⋯*A*	*D*—H⋯*A*
O2—H14⋯O1^i^	1.063 (18)	1.524 (18)	2.5856 (13)	176.8 (16)
C19—H10⋯N2^ii^	0.958 (16)	2.699 (16)	3.5991 (18)	156.8 (13)
C19—H11⋯O1^iii^	0.957 (17)	2.623 (17)	3.5518 (17)	163.7 (13)

Symmetry codes: (i) 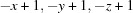; (ii) 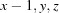; (iii) 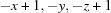.

## supplementary crystallographic information

### S1. Structural commentary

Pheno­thia­zine, C_12_H_9_NS showed stable redox properties. This crystal struture have been reported for the neutral state (Bell *et al.*, 1968, and van de Wall & Feli, 1977), and for the C_12_H_9_NS+ radical cation state (Sun *et al.*, 2004), and pheno­thia­zine-picric acid (1/1) (Harrison *et al.*, 2007).

As part of our studies for understanding the donor-acceptor inter­action type dye molecule for dye-sensitized solar cell (Watanabe *et al.*, 2014), we now report the title compound. In the title compound, C_21_H_14_N_2_O_2_S, consists a 12-methyl-12*H*-benzo[*b*]pheno­thia­zine as a donor moiety, which has a (*E*)-2-cyano­but-2-enoic acid moiety as acceptor at C-11 position of 12-methyl-12*H*-benzo[*b*]pheno­thia­zine.

Dark-red crystals were obtained from 0.013 g of title compound in tetra­hydro­furan (10 ml) solution by slow diffusion.

The C_21_H_14_N_2_O_2_S of 12-methyl-12*H*-benzo[*b*]pheno­thia­zine moiety takes pyramidal structure, in which benzene and naphthalene had 147.2°, while the olefin bond at the cyano­acrylic moiety existed 123.8° from the naphthalene plane (Figure 1). The title compounds showed an inter­molecular hydrogen bonding at O1—H14 - O2' (1.520 Å) in the two molecule structure of carb­oxy­lic acid moiety (Figure 2). Furthermore, title compound have π-π stacking orientation along with *a* axis, where the two-naphthalene moieties oriented the parallel structures, which has a transannular distance at 3.273 Å (Figure 3).

### S2. Crystallization

The chloro­form solution (10 ml) of title compound (0.013 g) was standing under ambient condition until the desired single crystal was produced.

### S3. Refinement

Crystal data, data collection and structure refinement details are summarized in Table 1. All the hydrogen atoms of the compound are fixed geometrically (C—H = 0.95 - 1.01 Å) and allowed to ride on their parent atoms. Structure was refined with unique reflections and with a cut-off sigma = 2.00.

### Crystal data

**Table:**

C_21_H_14_N_2_O_2_S	*F*(000) = 372
*M**_r_* = 358.40	*D*_x_ = 1.402 Mg m^−^^3^
Triclinic, *P*1	Melting point: 351 K
*a* = 6.7915 (17) Å	Mo *K*α radiation, λ = 0.71075 Å
*b* = 9.196 (3) Å	Cell parameters from 14164 reflections
*c* = 13.941 (3) Å	θ = 3.0–27.5°
α = 95.831 (13)°	µ = 0.21 mm^−^^1^
β = 101.214 (10)°	*T* = 123 K
γ = 90.850 (15)°	Block, dark red
*V* = 849.1 (4) Å^3^	0.40 × 0.40 × 0.15 mm
*Z* = 2	

### Data collection

**Table:**

Rigaku R-AXIS RAPID CCD diffractometer	*R*_int_ = 0.020
ω/2θ scans	θ_max_ = 27.5°, θ_min_ = 3.0°
14164 measured reflections	*h* = −8→8
3877 independent reflections	*k* = −11→11
3663 reflections with *I* > 2σ(*I*)	*l* = −18→16

### Refinement

**Table:**

Refinement on *F*^2^	0 restraints
Least-squares matrix: full	Hydrogen site location: difference Fourier map
*R*[*F*^2^ > 2σ(*F*^2^)] = 0.036	H-atom parameters constrained
*wR*(*F*^2^) = 0.100	*w* = 1/[σ^2^(*F*_o_^2^) + (0.0609*P*)^2^ + 0.2744*P*] where *P* = (*F*_o_^2^ + 2*F*_c_^2^)/3
*S* = 1.06	(Δ/σ)_max_ < 0.001
3877 reflections	Δρ_max_ = 0.29 e Å^−^^3^
290 parameters	Δρ_min_ = −0.40 e Å^−^^3^

### Special details

**Table:**

Geometry. All e.s.d.'s (except the e.s.d. in the dihedral angle between two l.s. planes) are estimated using the full covariance matrix. The cell e.s.d.'s are taken into account individually in the estimation of e.s.d.'s in distances, angles and torsion angles; correlations between e.s.d.'s in cell parameters are only used when they are defined by crystal symmetry. An approximate (isotropic) treatment of cell e.s.d.'s is used for estimating e.s.d.'s involving l.s. planes.

### Fractional atomic coordinates and isotropic or equivalent isotropic displacement parameters (Å^2^)

**Table:**

	*x*	*y*	*z*	*U*_iso_*/*U*_eq_	
H2	0.953 (3)	−0.333 (2)	0.4654 (13)	0.041 (5)*	
H3	0.968 (3)	−0.526 (2)	0.3462 (15)	0.056 (6)*	
C1	0.71497 (19)	−0.23569 (15)	0.38906 (10)	0.0297 (3)	
H10	0.213 (2)	−0.0357 (18)	0.3396 (12)	0.029 (4)*	
C2	0.8586 (2)	−0.34194 (17)	0.40418 (12)	0.0371 (3)	
C3	0.8644 (2)	−0.45592 (16)	0.33152 (13)	0.0402 (3)	
C4	0.7256 (2)	−0.46500 (15)	0.24404 (12)	0.0353 (3)	
C5	0.12589 (17)	0.17296 (15)	−0.11405 (9)	0.0274 (3)	
H5	0.290 (2)	−0.2245 (18)	−0.0436 (12)	0.033 (4)*	
H6	0.163 (2)	−0.0447 (18)	−0.1546 (12)	0.031 (4)*	
H9	0.238 (2)	0.3227 (18)	0.1163 (12)	0.031 (4)*	
C6	0.22142 (17)	0.24648 (13)	0.06076 (9)	0.0223 (2)	
C7	0.34176 (15)	0.06021 (12)	0.17662 (8)	0.0173 (2)	
S1	0.39308 (5)	−0.37579 (3)	0.12102 (2)	0.02878 (11)	
O1	0.65112 (12)	0.36226 (9)	0.47776 (6)	0.02296 (18)	
H7	0.075 (3)	0.1979 (19)	−0.1817 (13)	0.038 (4)*	
H1	0.714 (2)	−0.1535 (19)	0.4397 (12)	0.029 (4)*	
H8	0.124 (3)	0.386 (2)	−0.0451 (13)	0.040 (4)*	
C8	0.24793 (16)	−0.00669 (13)	−0.00306 (8)	0.0202 (2)	
H4	0.725 (3)	−0.541 (2)	0.1909 (13)	0.041 (5)*	
C9	0.26789 (15)	0.10113 (12)	0.07897 (8)	0.0181 (2)	
O2	0.35247 (13)	0.41863 (10)	0.39083 (6)	0.0275 (2)	
H14	0.353 (3)	0.507 (2)	0.4467 (13)	0.041*	
N1	0.42827 (14)	−0.13630 (10)	0.28349 (7)	0.0208 (2)	
C10	0.38246 (15)	−0.08478 (12)	0.19057 (8)	0.0177 (2)	
H13	0.246 (2)	0.2398 (16)	0.2575 (11)	0.023 (3)*	
C11	0.35977 (16)	0.17829 (12)	0.25662 (8)	0.0186 (2)	
C12	0.50856 (16)	0.34153 (12)	0.40510 (8)	0.0185 (2)	
N2	0.86564 (16)	0.10083 (13)	0.34623 (9)	0.0336 (3)	
H11	0.312 (2)	−0.1618 (18)	0.3995 (12)	0.031 (4)*	
H12	0.420 (3)	−0.003 (2)	0.4129 (13)	0.041 (5)*	
C13	0.30285 (16)	−0.15078 (13)	0.01314 (8)	0.0219 (2)	
C14	0.36473 (16)	−0.18954 (12)	0.10592 (8)	0.0201 (2)	
C15	0.17442 (17)	0.03230 (15)	−0.09906 (8)	0.0250 (2)	
C16	0.15205 (18)	0.28132 (14)	−0.03332 (9)	0.0266 (3)	
C17	0.57900 (18)	−0.36047 (13)	0.22895 (10)	0.0262 (2)	
C18	0.57427 (17)	−0.24412 (12)	0.30077 (9)	0.0231 (2)	
C19	0.33548 (19)	−0.08052 (14)	0.36491 (9)	0.0248 (2)	
C20	0.51890 (16)	0.21732 (12)	0.32950 (8)	0.0186 (2)	
C21	0.70959 (17)	0.14883 (13)	0.33855 (8)	0.0222 (2)	

### Atomic displacement parameters (Å^2^)

**Table:**

	*U*^11^	*U*^22^	*U*^33^	*U*^12^	*U*^13^	*U*^23^
C1	0.0244 (6)	0.0300 (6)	0.0354 (7)	0.0032 (5)	0.0037 (5)	0.0107 (5)
C2	0.0239 (6)	0.0388 (7)	0.0513 (8)	0.0065 (5)	0.0052 (6)	0.0218 (6)
C3	0.0290 (6)	0.0339 (7)	0.0655 (10)	0.0136 (5)	0.0183 (6)	0.0231 (7)
C4	0.0344 (7)	0.0229 (6)	0.0559 (9)	0.0083 (5)	0.0227 (6)	0.0106 (6)
C5	0.0187 (5)	0.0433 (7)	0.0200 (5)	−0.0067 (5)	0.0017 (4)	0.0078 (5)
C6	0.0189 (5)	0.0240 (6)	0.0231 (5)	−0.0022 (4)	0.0023 (4)	0.0017 (4)
C7	0.0140 (4)	0.0198 (5)	0.0168 (5)	−0.0007 (4)	0.0021 (4)	−0.0026 (4)
S1	0.03269 (18)	0.01645 (16)	0.03660 (19)	−0.00299 (11)	0.00977 (13)	−0.00517 (11)
O1	0.0245 (4)	0.0230 (4)	0.0180 (4)	0.0017 (3)	−0.0007 (3)	−0.0045 (3)
C8	0.0143 (4)	0.0278 (6)	0.0176 (5)	−0.0054 (4)	0.0043 (4)	−0.0026 (4)
C9	0.0130 (4)	0.0223 (5)	0.0177 (5)	−0.0028 (4)	0.0019 (4)	−0.0009 (4)
O2	0.0262 (4)	0.0258 (4)	0.0262 (4)	0.0094 (3)	−0.0004 (3)	−0.0080 (3)
N1	0.0208 (4)	0.0211 (5)	0.0207 (4)	0.0053 (4)	0.0046 (4)	0.0015 (3)
C10	0.0137 (4)	0.0198 (5)	0.0186 (5)	−0.0008 (4)	0.0034 (4)	−0.0020 (4)
C11	0.0195 (5)	0.0178 (5)	0.0179 (5)	0.0024 (4)	0.0034 (4)	−0.0003 (4)
C12	0.0197 (5)	0.0177 (5)	0.0170 (5)	0.0013 (4)	0.0028 (4)	−0.0012 (4)
N2	0.0236 (5)	0.0391 (6)	0.0338 (6)	0.0074 (4)	0.0017 (4)	−0.0098 (5)
C13	0.0183 (5)	0.0251 (6)	0.0210 (5)	−0.0056 (4)	0.0065 (4)	−0.0083 (4)
C14	0.0174 (5)	0.0178 (5)	0.0247 (5)	−0.0024 (4)	0.0065 (4)	−0.0042 (4)
C15	0.0176 (5)	0.0392 (7)	0.0171 (5)	−0.0080 (4)	0.0042 (4)	−0.0018 (5)
C16	0.0204 (5)	0.0320 (6)	0.0276 (6)	−0.0026 (4)	0.0024 (4)	0.0089 (5)
C17	0.0241 (5)	0.0197 (5)	0.0381 (6)	0.0017 (4)	0.0131 (5)	0.0055 (5)
C18	0.0191 (5)	0.0209 (5)	0.0312 (6)	0.0027 (4)	0.0077 (4)	0.0066 (4)
C19	0.0270 (6)	0.0275 (6)	0.0218 (5)	0.0055 (5)	0.0086 (4)	0.0035 (4)
C20	0.0197 (5)	0.0177 (5)	0.0176 (5)	0.0020 (4)	0.0034 (4)	−0.0026 (4)
C21	0.0218 (5)	0.0226 (5)	0.0195 (5)	0.0005 (4)	0.0018 (4)	−0.0064 (4)

### Geometric parameters (Å, º)

**Table:**

C1—C2	1.3933 (18)	C8—C15	1.4185 (16)
C1—C18	1.3982 (18)	C8—C9	1.4192 (15)
C1—H1	0.982 (17)	O2—C12	1.2786 (14)
C2—C3	1.388 (2)	O2—H14	1.063 (18)
C2—H2	0.959 (18)	N1—C10	1.4042 (14)
C3—C4	1.383 (2)	N1—C18	1.4174 (14)
C3—H3	0.97 (2)	N1—C19	1.4553 (14)
C4—C17	1.3956 (17)	C10—C14	1.4301 (15)
C4—H4	0.963 (19)	C11—C20	1.3478 (15)
C5—C15	1.366 (2)	C11—H13	0.964 (15)
C5—C16	1.4072 (19)	C12—C20	1.4855 (15)
C5—H7	0.990 (17)	N2—C21	1.1440 (16)
C6—C16	1.3764 (17)	C13—C14	1.3650 (17)
C6—C9	1.4145 (16)	C13—H5	0.978 (17)
C6—H9	0.978 (17)	C15—H6	0.985 (17)
C7—C10	1.3908 (16)	C16—H8	1.006 (18)
C7—C9	1.4458 (15)	C17—C18	1.3942 (18)
C7—C11	1.4611 (15)	C19—H10	0.958 (16)
S1—C14	1.7556 (13)	C19—H11	0.957 (17)
S1—C17	1.7581 (14)	C19—H12	1.013 (19)
O1—C12	1.2544 (14)	C20—C21	1.4376 (15)
C8—C13	1.4101 (17)		
			
C2—C1—C18	120.09 (13)	C20—C11—C7	128.50 (10)
C2—C1—H1	120.2 (9)	C20—C11—H13	114.1 (9)
C18—C1—H1	119.7 (9)	C7—C11—H13	117.3 (9)
C3—C2—C1	120.34 (14)	O1—C12—O2	125.12 (10)
C3—C2—H2	121.5 (11)	O1—C12—C20	118.13 (10)
C1—C2—H2	118.2 (11)	O2—C12—C20	116.75 (9)
C4—C3—C2	119.87 (12)	C14—C13—C8	121.34 (10)
C4—C3—H3	123.7 (12)	C14—C13—H5	120.0 (10)
C2—C3—H3	116.5 (12)	C8—C13—H5	118.6 (10)
C3—C4—C17	120.17 (14)	C13—C14—C10	121.42 (10)
C3—C4—H4	123.1 (11)	C13—C14—S1	118.47 (8)
C17—C4—H4	116.8 (11)	C10—C14—S1	119.66 (9)
C15—C5—C16	119.59 (11)	C5—C15—C8	120.96 (11)
C15—C5—H7	119.6 (10)	C5—C15—H6	121.2 (9)
C16—C5—H7	120.8 (10)	C8—C15—H6	117.8 (9)
C16—C6—C9	121.02 (11)	C6—C16—C5	120.73 (12)
C16—C6—H9	119.9 (9)	C6—C16—H8	119.9 (10)
C9—C6—H9	119.1 (9)	C5—C16—H8	119.4 (10)
C10—C7—C9	120.36 (9)	C18—C17—C4	120.36 (13)
C10—C7—C11	123.92 (10)	C18—C17—S1	118.84 (9)
C9—C7—C11	115.66 (10)	C4—C17—S1	120.77 (11)
C14—S1—C17	99.34 (6)	C17—C18—C1	119.14 (11)
C13—C8—C15	121.48 (10)	C17—C18—N1	119.89 (11)
C13—C8—C9	118.85 (10)	C1—C18—N1	120.97 (11)
C15—C8—C9	119.67 (11)	N1—C19—H10	109.2 (9)
C6—C9—C8	117.97 (10)	N1—C19—H11	107.3 (10)
C6—C9—C7	122.69 (10)	H10—C19—H11	111.2 (14)
C8—C9—C7	119.29 (10)	N1—C19—H12	114.0 (10)
C12—O2—H14	113.7 (10)	H10—C19—H12	106.4 (14)
C10—N1—C18	119.48 (9)	H11—C19—H12	108.7 (14)
C10—N1—C19	122.62 (9)	C11—C20—C21	123.98 (10)
C18—N1—C19	117.88 (9)	C11—C20—C12	120.73 (10)
C7—C10—N1	123.61 (9)	C21—C20—C12	115.25 (9)
C7—C10—C14	118.54 (10)	N2—C21—C20	176.72 (13)
N1—C10—C14	117.73 (10)		
			
C18—C1—C2—C3	0.6 (2)	C7—C10—C14—S1	−174.34 (8)
C1—C2—C3—C4	−0.7 (2)	N1—C10—C14—S1	1.83 (13)
C2—C3—C4—C17	−0.4 (2)	C17—S1—C14—C13	152.24 (9)
C16—C6—C9—C8	2.30 (16)	C17—S1—C14—C10	−35.38 (10)
C16—C6—C9—C7	179.77 (10)	C16—C5—C15—C8	0.99 (17)
C13—C8—C9—C6	176.94 (9)	C13—C8—C15—C5	−178.54 (10)
C15—C8—C9—C6	−2.82 (15)	C9—C8—C15—C5	1.22 (16)
C13—C8—C9—C7	−0.62 (15)	C9—C6—C16—C5	−0.12 (18)
C15—C8—C9—C7	179.61 (9)	C15—C5—C16—C6	−1.56 (18)
C10—C7—C9—C6	179.09 (9)	C3—C4—C17—C18	1.62 (19)
C11—C7—C9—C6	1.88 (15)	C3—C4—C17—S1	−176.53 (10)
C10—C7—C9—C8	−3.47 (15)	C14—S1—C17—C18	36.16 (10)
C11—C7—C9—C8	179.32 (9)	C14—S1—C17—C4	−145.67 (10)
C9—C7—C10—N1	−171.10 (9)	C4—C17—C18—C1	−1.66 (17)
C11—C7—C10—N1	5.86 (16)	S1—C17—C18—C1	176.53 (9)
C9—C7—C10—C14	4.83 (15)	C4—C17—C18—N1	178.19 (11)
C11—C7—C10—C14	−178.20 (9)	S1—C17—C18—N1	−3.63 (15)
C18—N1—C10—C7	−142.33 (11)	C2—C1—C18—C17	0.53 (18)
C19—N1—C10—C7	35.94 (16)	C2—C1—C18—N1	−179.31 (11)
C18—N1—C10—C14	41.71 (14)	C10—N1—C18—C17	−41.32 (15)
C19—N1—C10—C14	−140.02 (11)	C19—N1—C18—C17	140.33 (11)
C10—C7—C11—C20	53.51 (17)	C10—N1—C18—C1	138.52 (11)
C9—C7—C11—C20	−129.39 (12)	C19—N1—C18—C1	−39.83 (16)
C15—C8—C13—C14	−176.93 (10)	C7—C11—C20—C21	2.65 (19)
C9—C8—C13—C14	3.31 (16)	C7—C11—C20—C12	−179.85 (10)
C8—C13—C14—C10	−1.94 (16)	O1—C12—C20—C11	171.53 (10)
C8—C13—C14—S1	170.30 (8)	O2—C12—C20—C11	−7.97 (16)
C7—C10—C14—C13	−2.19 (15)	O1—C12—C20—C21	−10.77 (15)
N1—C10—C14—C13	173.98 (10)	O2—C12—C20—C21	169.73 (10)

### Hydrogen-bond geometry (Å, º)

**Table:**

*D*—H···*A*	*D*—H	H···*A*	*D*···*A*	*D*—H···*A*
O2—H14···O1^i^	1.063 (18)	1.524 (18)	2.5856 (13)	176.8 (16)
C19—H10···N2^ii^	0.958 (16)	2.699 (16)	3.5991 (18)	156.8 (13)
C19—H11···O1^iii^	0.957 (17)	2.623 (17)	3.5518 (17)	163.7 (13)

Symmetry codes: (i) −*x*+1, −*y*+1, −*z*+1; (ii) *x*−1, *y*, *z*; (iii) −*x*+1, −*y*, −*z*+1.
