# Supplementary figures and images for: Crystal structure of (E)-2-cyano-3-(12-methyl-12H-benzo[b]pheno­thia­zin-11-yl)acrylic acid
Source: Acta Crystallogr Sect E Struct Rep Online. 2014 Aug 20;70(Pt 9):o1026–7. doi: 10.1107/S1600536814018388 (PMC4186111; doi:10.1107/S1600536814018388)

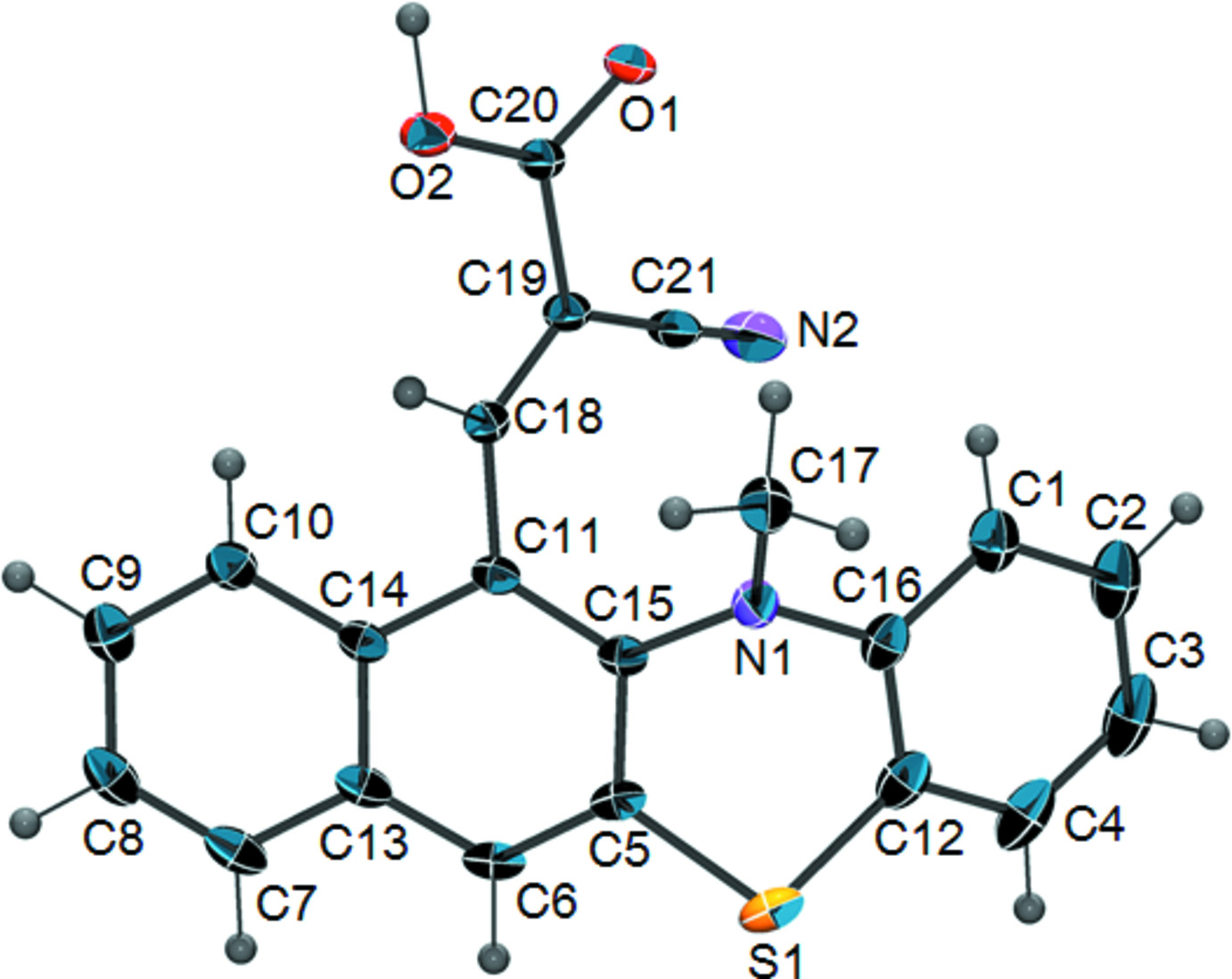

Supplement: Supplementary file 4 [file e-70-o1026-fig1.tif]

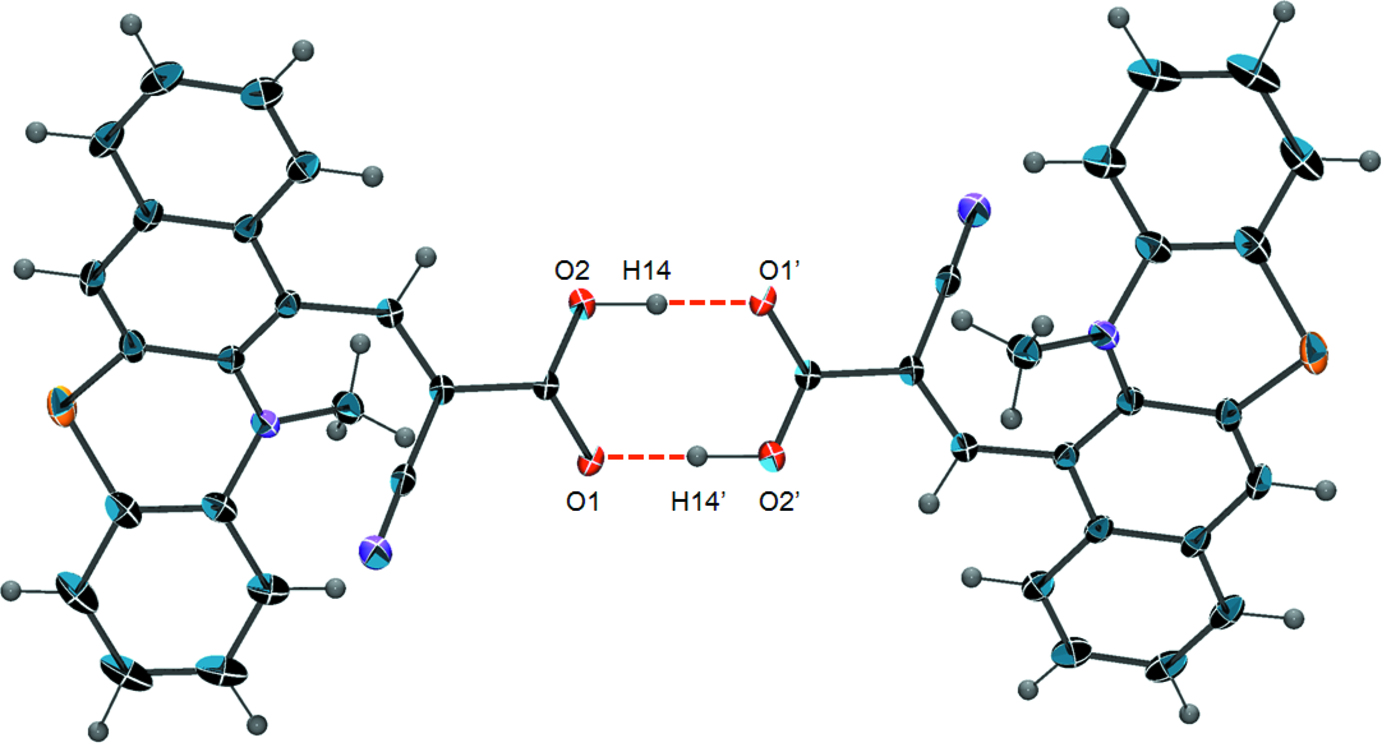

Supplement: Supplementary file 5 [file e-70-o1026-fig2.tif]

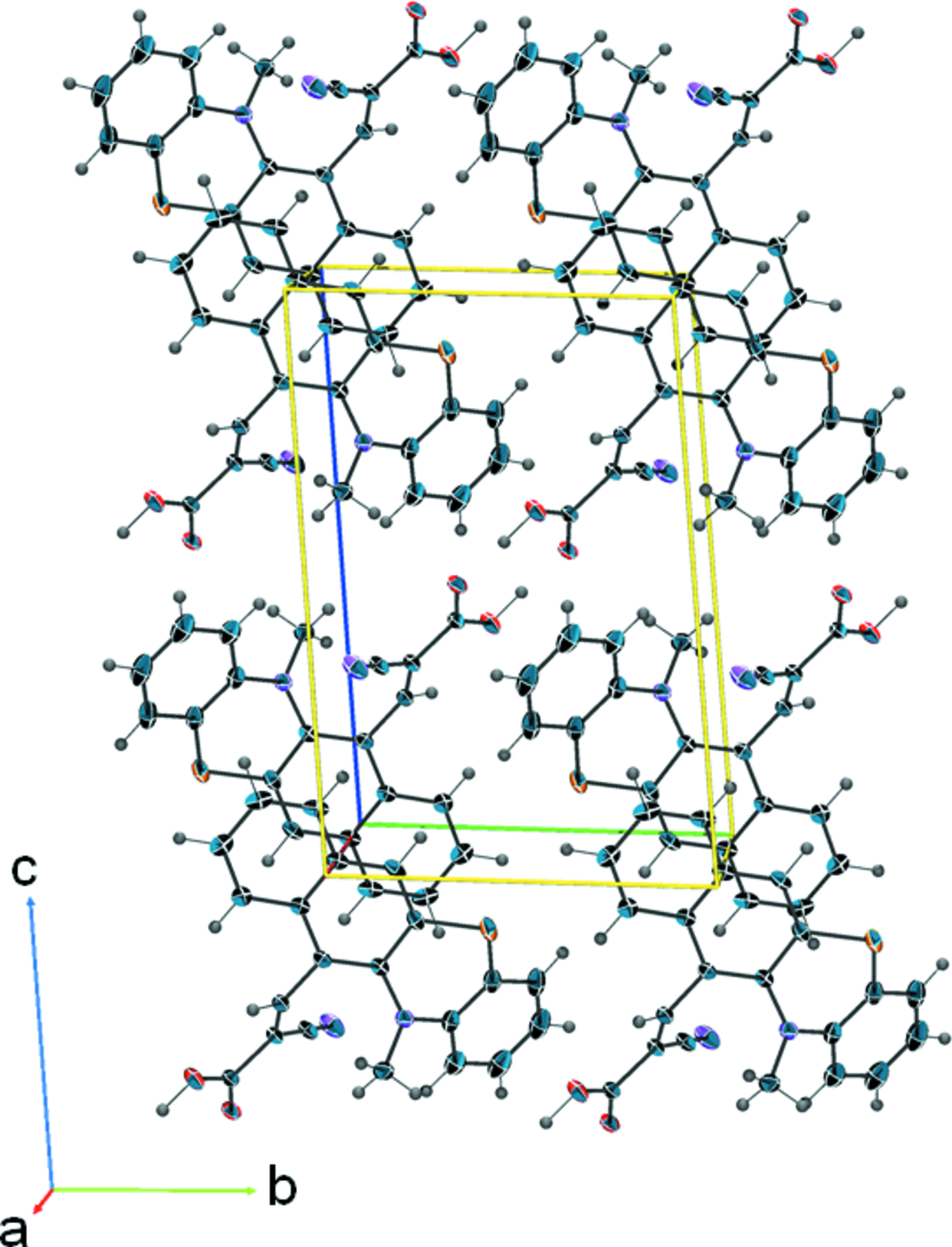

Supplement: Supplementary file 6 [file e-70-o1026-fig3.tif]
